# Supplementary material for: Family context and individual characteristics in antenatal care utilization among adolescent childbearing mothers in urban slums in Nigeria
Source: PLoS One. 2021 Nov 29;16(11):e0260588. doi: 10.1371/journal.pone.0260588 (PMC8629214; doi:10.1371/journal.pone.0260588)
Supplement: Attachment — Submitted filename: rebuttal letter.pdf [file pone.0260588.s003.pdf]

The Academic Editor,
PLOS ONE.

October 13th , 2021.

Response to Editor's/Reviewers' Comments

We sincerely wish to thank the Editor and the Reviewers for the comments and suggestions. We have attended to all the issues.

Minor essential comments

**Comment Reviewer 1**	**Response**
The Introduction section should provide more insights on the situation in Nigeria urban slums, considering the focus of the paper. Information on average ANC participation in these areas, or on adolescent health in general could prove useful for the reader.	The introduction is reviewed to reflect this. The last paragraph of the introduction already contextualize the problem in urban slums in Nigeria. However, there is no evidence on ANC participation in slum areas in Nigeria.
The Measurement of Variables section should present a clear explanation of how the social status indicator has been calculated, considering its relevance in the findings.	reviewed
The role of wealth is not discussed in the Results/Discussion sections, although it is marginally significant. Results are also not reflective of previous literature on the role of wealth on ANC (i.e., the authors find that being wealthier is not correlated with a higher probability of attending 4ANC+). The authors should present hypotheses and potential explanations of their findings to explain why the situation in which households leave in the slums would reduce the importance of wealth in influencing access to ANC.	Reviewed to reflect this
**Comment Reviewer 2**	**Response**
**BACKGROUND**	
Line 6-6: "The greatest burden is concentrated in West and Central Africa where 6 per-cent of adolescents reported births before age 15 and 28 per cent of women between the ages of 20 and 24 reported a birth before age 18." The authors need to cite compared to what is the West and Central Africa have the greatest burden.	The sentence is now rephrased.

Line 26 -27: Research evidence from developing countries consistently underscores the importance of ANC among childbearing adolescents. What are some of the positive impacts of ANC for childbearing adolescents? For instance, what percent of death reduction is attributed to ANC?	Reviewed
Line 11-26: This section may need more argument why they have conducted their study. For instance, is there no study that examined the influence of family context in ANC uptake among childbearing adolescents in urban slums in Nigeria? If any, what are the methodological and knowledge gaps you wanted to fill on the existing evidence? This section may benefit from more professional and scientific critics of existing evidence to call out their paper's strong side.	I did not find any study that has linked family context with ANC in Nigerian urban slums.
**METHODS**	
Line 31-32: Were there any special ethical procedures followed for adolescents? Does Nigeria's Research Ethics Guideline allow to take direct consent from women less than 18 years of age? Any assent or witness used during consent taking?	Reviewed
How was slum area defined in this paper? Did you take slum residents based on a set of criteria or slum sites/areas in general? Please provide brief explanation about the slum areas you studied.	In line with UNHabitat definition
Line 38-39: Why did you decide to include those having at least a child less than 5 years? Why not less than 1, why not less than 3 years? Brief explanation if you have any reason for your inclusion criteria.	Experience suggest that the sample frame be more open in order to get adequate sample.
Page 5: The data collection was implemented between July and October 2018. Why did it take this much time for data collection alone or does the period include the wider study period including analysis and report writing?	Data was collected in 3 different States with a focus on each state per time. In Lagos and Oyo, data collection was between July 3 and August 6, while in Kaduna, data collection was between August 15 to October 9. In addition, data collection extended across all the locations because of callback to households where the selected persons were not immediately available for interviews
Ethical approval related information is repeated between pages 4 and 5.	Reviewed

	The duplication in the method section has been deleted
Page 5: Measurement of variables: WHO has recommended at least 8 visits for a positive pregnancy experience? You have cited the World Health Organization (2016) minimum recommendations but the cut off you applied is older. The 2016 recommendation is a minimum of 8 contacts. Did you consider this, or it is because Nigeria didn't start implementing the new recommendation? Why did you decide a minimum of 4 visits rather than 8? Unless you justify this, you may need to re-analyze in the whole of the paper.	Reviewed
**RESULT**	
Page 6, Line 17-25…the comparisons between the current study and DHS is not in the right place. Better move it to the Discussion part.	DHS evidence deleted from the table
Line 29-38: I do not see the importance of presenting this section (the Bivariate analysis). It is repeated under the Binary; logistic regression model except the later shows net effects…Just explain how you used the bivariate analysis to select the variables entered to the multivariate model and focus on the multivariate findings.	Reviewed<br><br>Another reviewer recommended that the bivariate analyses are particularly important to the study and should be left as it is
Page 7, Line 7-24: The interpretation approach is not inviting for readers. I counted that the word "Those" was repeated 11 times in this section and this indicates that the authors need to be careful in articulating their findings. It needs to be re-written.	Reviewed
Page 7, Line 30-33: Authors have associated the influence of polygamous marriage in the North to 4+ANC visits. The possible explanation is not clear. The authors need to describe how ANC visit is associated with polygamous marriage and it must be supported with evidence. Readers do not want to read the authors' hypotheses but their evidence-based explanations. Plus, the "RESULT" section is not the right place to include possible explanations. The result section should only cover the findings in a simple language. Possible explanations and further interpretations of findings	I did not find any study linking ANC to polygamous marriage

should be addressed under the Discussion part of the paper.	
**DISCUSSION**	
The first paragraph on page 9 (mental health) issue seems out of context. Mental health was never mentioned in the previous sections.	Reviewed
It would be advisable if the authors could come up with an overall comprehensive conclusion at the end of the discussion part. Recommendations are already mixed in the discussion.	Reviewed and conclusion added
**Other Comments**	**Response**
PLOS ONE endorses the strobe statement checklist for cross-sectional studies, https://www.strobe-statement.org/fileadmin/Strobe/uploads/checklists/STROBE_checklist_v4_cross-sectional.pdf. You can use it as a guide in your revision since many of the comments of the reviewers' address issues from the checklist lacking in some aspects. Among these for instance, is the selection of participants (you mention "randomly", but this is vague: based on what? Did you have a listing? ...), how did you deal with missing data, participants (section 13), ...	Reviewed.
A second reservation is data availability. PLOS ONE policy is that authors are required to make all data underlying the findings described fully available, without restriction, and from the time of publication. PLOS allows rare exceptions to address legal and ethical concerns. See the PLOS Data Policy and FAQ for detailed information. This is not currently the case. It is wrongly stated that "All relevant data are within the manuscript and/or its Supporting Information files". This is not true. Let me remind you the possibilities:<br><br>➢ If the data are held or will be held in a public repository, include URLs, accession numbers or DOIs. If this information will only be available after acceptance, indicate this by ticking the box below. For example: All XXX files are available from	Data available upon request

the XXX database (accession number(s) XXX, XXX.).<br><br>➢ If the micro data are all contained within the manuscript and/or Supporting Information files, enter the following: All relevant data are within the manuscript and its Supporting Information files.<br><br>➢ If neither of these applies but you can provide details of access elsewhere, with or without limitations, please do so	
Please also include the questionnaire used in the study.	Included
In table 2 you should indicate within the columns that the comparison is not "fair" in the sense that DHS refers to the whole state, as I understand, and not to the urban slums. You can be more explicit in the header, probably include Slums Adolescent Survey vs State-level DHS.	Reviewed. DHS analysis deleted
I think the bivariate analysis is useful. Do not exclude it.	Not excluded
Regarding the inclusion of 8 visits, you can include it in the analysis if you like, otherwise, admitting the change in recommendation from 4 to 8 visits, you can argue why it is still interesting to look at 4 visits.	Reviewed
While you must improve the description of the variables retained for analysis (items 7 and 8 of strobe statement), this is particularly important regarding the wealth variables, as manifested by the reviewers. Are you defining quintiles based on this questionnaire or are you using wealth cuts according to the DHS? That would make results more comparable. Since only slum areas are included. Also, social status is not described in enough detail to allow for replication. There could also be a high overlap between wealth and social status, in which case that could explain non-significance in the multivariate analysis and high significance in the bivariate analysis.	Reviewed

Regarding the regression, you should include group tests of significance in table 4 and 5, since the tests for categorical variables that you report (individual) are less interesting since they compare to the reference category.	Added
Note also PLOS ONE guidelines for reporting statistical results, https://journals.plos.org/plosone/s/submission-guidelines#loc-statistical-reporting. In particular<br><br>• **Regression analyses.** Include the full results of any regression analysis performed as a supplementary file. Include all estimated regression coefficients, their standard error, p-values, and confidence intervals, as well as the measures of goodness of fit.<br>• **Reporting parameters.** Test statistics (F/t/r) and associated degrees of freedom should be provided. Effect sizes and confidence intervals should be reported where appropriate. If percentages are provided, the numerator and denominator should also be given.<br><br>**P-values.** Report exact p-values for all values greater than or equal to 0.001. P-values less than 0.001 may be expressed as $p < 0.001$, or as exponentials in studies of genetic associations.	Included as Appendix
**OTHER ISSUES 2**	
Please ensure that your manuscript meets PLOS ONE's style requirements, including those for file naming. The PLOS ONE style templates can be found at https://journals.plos.org/plosone/s/file?id=wjVg/PLOSOne_formatting_sample_main_body.pdf and https://journals.plos.org/plosone/s/file?id=ba62/PLOSOne_formatting_sample_title_authors_affiliations.pdf	
Please review your reference list to ensure that it is complete and correct. If you have cited papers that have been retracted, please include the rationale	

for doing so in the manuscript text or remove these references and replace them with relevant current references. Any changes to the reference list should be mentioned in the rebuttal letter that accompanies your revised manuscript. If you need to cite a retracted article, indicate the article's retracted status in the References list and also include a citation and full reference for the retraction notice.	
Please include additional information regarding the survey or questionnaire used in the study and ensure that you have provided sufficient details that others could replicate the analyses. For instance, if you developed a questionnaire as part of this study and it is not under a copyright more restrictive than CC-BY, please include a copy, in both the original language and English, as Supporting Information.	
We note that Figure 1 in your submission contains map images which may be copyrighted.<br>All PLOS content is published under the Creative Commons Attribution License (CC BY 4.0), which means that the manuscript, images, and Supporting Information files will be freely available online, and any third party is permitted to access, download, copy, distribute, and use these materials in any way, even commercially, with proper attribution. For these reasons, we cannot publish previously copyrighted maps or satellite images created using proprietary data, such as Google software (Google Maps, Street View, and Earth). For more information, see our copyright guidelines: http://journals.plos.org/plosone/s/licenses-and-copyright.<br><br>We require you to either (a) present written permission from the copyright holder to publish this figure specifically under the CC BY 4.0 license, or (b) remove the figure from your submission:<br>a. You may seek permission from the original copyright holder of Figure 1 to publish the content specifically under the CC BY 4.0 license.	

We recommend that you contact the original copyright holder with the Content Permission Form (http://journals.plos.org/plosone/s/file?id=7c09/content-permission-form.pdf) and the following text:<br>"I request permission for the open-access journal PLOS ONE to publish XXX under the Creative Commons Attribution License (CCAL) CC BY 4.0 (http://creativecommons.org/licenses/by/4.0/). Please be aware that this license allows unrestricted use and distribution, even commercially, by third parties. Please reply and provide explicit written permission to publish XXX under a CC BY license and complete the attached form."<br><br>Please upload the completed Content Permission Form or other proof of granted permissions as an "Other" file with your submission.<br><br>In the figure caption of the copyrighted figure, please include the following text: "Reprinted from [ref] under a CC BY license, with permission from [name of publisher], original copyright [original copyright year]."<br><br>b. If you are unable to obtain permission from the original copyright holder to publish this figure under the CC BY 4.0 license or if the copyright holder's requirements are incompatible with the CC BY 4.0 license, please either i) remove the figure or ii) supply a replacement figure that complies with the CC BY 4.0 license. Please check copyright information on all replacement figures and update the figure caption with source information. If applicable, please specify in the figure caption text when a figure is similar but not identical to the original image and is therefore for illustrative purposes only.<br>The following resources for replacing copyrighted map figures may be helpful:<br>USGS National Map Viewer (public domain): http://viewer.nationalmap.gov/viewer/<br>The Gateway to Astronaut Photography of Earth (public domain): http://eol.jsc.nasa.gov/sseop/clickmap/	

Maps at the CIA (public domain): https://www.cia.gov/library/publications/the-world-factbook/index.html and https://www.cia.gov/library/publications/cia-maps-publications/index.html<br>NASA Earth Observatory (public domain): http://earthobservatory.nasa.gov/<br>Landsat: http://landsat.visibleearth.nasa.gov/<br>USGS EROS (Earth Resources Observatory and Science (EROS) Center) (public domain): http://eros.usgs.gov/#<br>Natural Earth (public domain): http://www.naturalearthdata.com/	
Please include a separate caption for each figure in your manuscript.	
Please include your tables as part of your main manuscript and remove the individual files. Please note that supplementary tables should be uploaded as separate "supporting information" files.	Reviewed
We note that Figure 1 also includes an image of individuals.<br>As per the PLOS ONE policy (http://journals.plos.org/plosone/s/submission-guidelines#loc-human-subjects-research) on papers that include identifying, or potentially identifying, information, the individual(s) or parent(s)/guardian(s) must be informed of the terms of the PLOS open-access (CC-BY) license and provide specific permission for publication of these details under the terms of this license.<br>Please download the Consent Form for Publication in a PLOS Journal (http://journals.plos.org/plosone/s/file?id=8ce6/plos-consent-form-english.pdf).<br>The signed consent form should not be submitted with the manuscript but should be securely filed in the individual's case notes.<br>Please amend the methods section and ethics statement of the manuscript to explicitly state that the patient/participant has provided consent for publication: “The individual in this manuscript has	

given written informed consent (as outlined in PLOS consent form) to publish these case details". If you are unable to obtain consent from the subject of the photograph, you will need to remove the figure and any other textual identifying information or case descriptions for this individual,	

Thank you.
Sincerely,

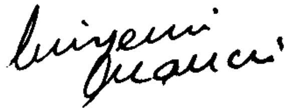

Prof. Akanni Akinyemi
