# Supplementary material for: Family context and individual characteristics in antenatal care utilization among adolescent childbearing mothers in urban slums in Nigeria
Source: PLoS One. 2021 Nov 29;16(11):e0260588. doi: 10.1371/journal.pone.0260588 (PMC8629214; doi:10.1371/journal.pone.0260588)
Supplement: Attachment — Submitted filename: Reviewer feedback.docx [file pone.0260588.s002.docx]

Reviewer feedback

**BACKGROUND**

Line 6-6: “The greatest burden is concentrated in West and Central Africa where 6 per-cent of adolescents reported births before age 15 and 28 per cent of women between the ages of 20 and 24 reported a birth before age 18.” The authors need to cite compared to what is the West and Central Africa have the greatest burden.

Line 26 -27: Research evidence from 26 developing countries consistently underscores the importance of ANC among childbearing 27 adolescents. What are some of the positive impacts of ANC for childbearing adolescents? For instance, what percent of death reduction is attributed to ANC?

Line 11-26: This section may need more argument why they have conducted their study. For instance, is there no study that examined the influence of family context in ANC uptake among childbearing adolescents in urban slums in Nigeria? If any, what are the methodological and knowledge gaps you wanted to fill on the existing evidence? This section may benefit from more professional and scientific critics of existing evidences to call out their paper’s strong side.

**METHODS**

Line 31-32: Were there any special ethical procedures followed for adolescents? Does Nigeria’s Research Ethics Guideline allow to take direct consent from women less than 18 years of age? Any assent or witness used during consent taking?

How was slum area defined in this paper? Did you take slum residents based on a set of criteria or slum sites/areas in general? Please provide brief explanation about the slum areas you studied.

Line 38-39: Why did you decide to include those having at least a child less than 5 years? Why not less than 1, why not less than 3 years? Brief explanation if you have any reason for your inclusion criteria.

Page 5: The data collection was implemented between July and October 2018. Why did it take this much time for data collection alone or does the period include the wider study period including analysis and report writing?

Ethical approval related information is repeated between pages 4 and 5.

Page 5: Measurement of variables: WHO has recommended at least 8 visits for a positive pregnancy experience? You have cited the World Health Organization (2016) minimum recommendations but the cut off you applied is older. The 2016 recommendation is a minimum of 8 contacts. Did you consider this or it is because Nigeria didn’t start implementing the new recommendation? Why did you decide a minimum of 4 visits rather than 8? Unless you justify this, you may need to re-analyze in the whole of the paper.

**RESULT**

Page 6, Line 17-25…the comparisons between the current study and DHS is not in the right place. Better move it to the Discussion part.

Line 29-38: I don’t see the importance of presenting this section (the Bivariate analysis). It is repeated under the Binary; logistic regression model except the later shows net effects…Just explain how you used the bivariate analysis to select the variables entered to the multivariate model and focus on the multivariate findings.

Page 7, Line 7-24: The interpretation approach is not inviting for readers. I counted that the word “Those” was repeated 11 times in this section and this indicates that the authors need to be careful in articulating their findings. It need to be re-written.

Page 7, Line 30-33: Authors have associated the influence of polygamous marriage in the North to 4+ANC visits. The possible explanation is not clear. The authors need to describe how ANC visit is associated with polygamous marriage and it has to be supported with evidence. Readers do not want to read the authors’ hypotheses but their evidence-based explanations. Plus, the “RESULT” section is not the right place to include possible explanations. The result section should only cover the findings in a simple language. Possible explanations and further interpretations of findings should be addressed under the Discussion part of the paper.

**DISCUSSION**

The first paragraph on page 9 (mental health) issue seems out of context. Mental health was never mentioned in the previous sections.

It would be advisable if the authors could come up with an overall comprehensive conclusion at the end of the discussion part. Recommendations are already mixed in the discussion.
